# From Composition to Acceptance: Linking Nutritional, Structural and Sensory Attributes in Clean-Label Breads

**DOI:** 10.3390/foods15050831

**Published:** 2026-03-02

**Authors:** Manuela Sanna, Stefano Sanna, Marco Serra, Tonina Roggio, Pasquale Catzeddu, Vanna Sanna

**Affiliations:** Porto Conte Ricerche, Loc. Tramariglio, 07041 Alghero, Italy; sanna@portocontericerche.it (M.S.); sannas@portocontericerche.it (S.S.); serram@portocontericerche.it (M.S.); roggio@portocontericerche.it (T.R.)

**Keywords:** clean label bread, physicochemical characterization, polyphenols, antioxidant activity, free amino acids, consumer perception, CATA method, sensory drivers of liking

## Abstract

The growing demand for clean-label bakery products requires a deeper understanding of how functional ingredients and physicochemical properties shape consumer perception. This study characterized nine commercial clean-label breads formulated with alternative flours, oilseeds, and functional ingredients by integrating instrumental analyses (color, porosity, free amino acids, total phenolic content, antioxidant activity) with consumer evaluation using hedonic testing and Check-All-That-Apply (CATA). Sixty-five consumers evaluated the breads under blind conditions. Results showed that flour type and seed inclusion significantly affected color, structure, and bioactive compound levels. Breads with higher phenolic content and antioxidant activity (GB-B, GB-C, GB-D, PB-I) exhibited more complex aroma profiles, whereas breads with higher porosity (GB-A, PB-G) were perceived as softer. Taste and texture showed the strongest correlation with overall liking (*r* > 0.84). CATA and penalty analysis identified soft, easy to chew, sweet, and umami as key drivers of liking, while dry, adhesive, bran odor, and bitter negatively impacted acceptance. Data revealed that consumer preference depends on the balance between structural attributes, flavor development, and nutritional composition. These findings provide actionable insights for the formulation of clean-label breads that balance health benefits and sensory acceptance.

## 1. Introduction

Bread is a fundamental and widely consumed foodstuff worldwide, with a millennia-old culinary tradition [1]. Its long, rich history has seen it evolve through countless forms, adopting various formulas, processes, and ingredients [2]. Yet, while bread remains a global staple, the baking industry is currently undergoing a phase of rapid innovation. This significant shift is not merely driven by technological or economic considerations; it is primarily influenced by growing consumer interest in health, well-being, and functional nutrition [3].

Health-conscious consumers increasingly seek foods that provide benefits beyond basic nutrition, favoring products that support well-being and disease prevention [4,5]. This demand has challenged the widespread use of highly milled, low-extraction wheat flour in conventional formulations, orienting research toward the development of enriched and functional breads [6].

Strategies to enhance the nutritional value of bread typically include increasing fiber content, essential for gut health and glycemic regulation [7,8], which is particularly beneficial for individuals following gluten-free diets, as these are often low in fiber. Other approaches involve improving the protein profile to meet the needs of specific population groups such as the elderly or vegetarians [9], and enhancing antioxidant activity through the incorporation of bioactive compounds to combat oxidative stress [10]. Furthermore, the enrichment of breads is recognized as an effective way to prevent nutrient deficiencies, given the product’s widespread consumption [11].

In recent years, these reformulation efforts have increasingly aligned with the principles of the “clean label” movement, which promotes the use of simple, minimally processed, and easily recognizable ingredients while limiting synthetic additives [12,13]. Although not scientifically defined, the clean-label concept is widely adopted by industry, consumers, and regulatory bodies, becoming a key driver in the development of new bakery products [14,15]. Within this framework, the use of alternative flours—derived from legumes, pseudocereals, ancient grains, or whole grains—and the incorporation of oilseeds have gained prominence. These ingredients contribute water-binding, film-forming, and nutritional properties that can enhance the functional profile of bread while meeting consumer expectations for natural and wholesome products [16,17,18].

While the nutritional benefits of enrichment are well-documented, the inclusion of non-wheat ingredients fundamentally modifies the dough matrix. Changes in protein composition, fiber content, and bioactive compounds influence key chemical pathways such as the Maillard reaction and alter structural attributes including porosity, volume, and crust and crumb color [19]. These modifications, in turn, affect the sensory properties of bread attributes that ultimately determine consumer acceptance and market success [20,21]. Visual appearance (color, porosity, seed distribution) plays a particularly influential role in shaping expectations of flavor and texture and in guiding purchase decisions [22,23,24]. Taste perception is also critical: free amino acids (FAA) contribute directly to sweetness, bitterness, and umami, and their concentration depends on flour composition and fermentation dynamics [25,26,27,28]. Understanding how these biochemical and physical parameters translate into sensory perception is therefore essential for designing functional breads that balance nutritional enhancement with consumer appeal.

Despite the rapid growth of research on enriched and innovative breads, the integration of detailed physicochemical characterization with consumer-based sensory evaluation remains largely underexplored [29,30].

Advances in sensory science now offer robust tools such as preference mapping, Check-All-That-Apply (CATA) analysis, and combined hedonic–descriptive modelling, which enable researchers to link objective instrumental measurements with subjective consumer perception in a comprehensive and interpretable manner [30,31].

In particular, the CATA method has become a widely adopted approach for consumer profiling due to its simplicity, discriminative power, and ability to capture sensory attributes using consumer language. When combined with multivariate techniques such as correspondence analysis and penalty-lift analysis, CATA provides deep insights into the drivers of liking and the sensory consequences of formulation changes. These developments provide a comprehensive framework for interpreting complex consumer reactions to novel bread formulations and for identifying the sensory drivers of acceptance in functional foods.

In this context, the present study aims to comprehensively characterize a selection of commercial breads formulated according to clean-label principles and enriched with non-conventional ingredients. The objectives were to: (i) quantify and compare key physicochemical parameters, including color, porosity, FAA composition, total phenolic content, and antioxidant activity, and (ii) evaluate sensory acceptability and consumer perception, under blind conditions, through an integrated approach combining hedonic testing, CATA methodology, penalty-lift analysis, and multivariate statistical techniques.

## 2. Materials and Methods

### 2.1. Samples

A total of nine commercial sliced pan breads from Mulino Bianco (Barilla Group, Parma, Italy), all readily available in Italian supermarkets, were selected. We chose samples manufactured using the same process but different formulations (labeled Samples GB to P, see Table 1). The decision to include both refined and wholewheat varieties was a deliberate strategy to characterize the entire product space of a single commercial line. To ensure freshness, all samples had approximately the same expiration date. For chemical analyses, the bread samples were finely ground using a mixer to obtain a homogeneous powder and then frozen at −32 °C.

### 2.2. Nutritional Composition

The nutritional composition of bread samples, as reported on the product labels, is summarized in Table S1. The nutritional analysis reveals substantial variability in macronutrient composition, primarily driven by the diversity of ingredients and the flour extraction rate. While energy values remain relatively stable across samples (258 to 297 kcal/100 g), the nutrient profiles vary significantly, reflecting distinct formulation strategies.

Fat content emerged as the most variable component, ranging from 3.9 to 10 g/100 g. Samples GB-B, GB-C, GB-D, and PB-I exhibited the highest lipid levels (8–10 g/100 g), due to the inclusion of oilseeds, vegetable oils, or walnuts. Notably, PB-I was the only sample containing vitamin E (15 mg), consistent with the addition of flaxseed oil and the declared vitamin E fortification (0.03%).

Carbohydrate content ranged from 32.1 to 48.4 g/100 g, reflecting the flour extraction rate. Breads made with Type 0 flour (GB-A, PB-E) maintained a starch-rich matrix, whereas multigrain or enriched formulations (GB-D, PB-I) showed reduced carbohydrate density due to the partial substitution of flour with lipid- and protein-rich ingredients.

A clear relationship was observed between flour extraction rate and fiber content. Samples GB-D and PB-F, containing whole wheat flour as a primary ingredient (56.2% and 66.6% respectively), achieved the highest fiber concentrations (8.2–8.4 g/100 g). This aligns with established literature regarding the preservation of bran and germ layers in whole-grain products [32]. Conversely, low extraction flour breads (GB-A and PB-E) showed the lowest fiber values (4.0 g–4.5 g). Interestingly, GB-C and PB-I utilized “wheat fiber” as a separate additive to boost fiber levels despite being based on durum semolina or Type 0 flour.

Protein content (8.5 to 12 g/100 g) was strongly influenced by both the base flour type and supplementary protein sources. High-protein samples, specifically PB-G and PB-I, reached the highest levels through the incorporation of soy grits (5.1% and 4.3%, respectively) and wheat gluten. The use of soy is particularly significant as it introduces a legume-based protein source, potentially improving the amino acid profile of the final product [33]. Furthermore, GB-C benefited from the naturally higher protein content of durum wheat combined with added gluten, while the elevated protein in GB-D and PB-F samples was primarily due to the use of whole wheat flour.

Finally, salt content was relatively uniform across samples (0.8–1.3 g/100 g), consistent with typical bread formulations and regulatory guidelines aimed at balancing flavor and dough rheology [34].

### 2.3. Colour Analysis

The color parameters of different breads were measured using a CM-700d spectrophotometer (Konica Minolta Inc., Osaka, Japan) with a D65 illuminant and a 10° CIE standard observer. The analysis recorded the L* (lightness), a* (redness/greenness), and b* (yellowness/blueness) coordinates. Before measuring the crust and crumb color of each bread, the spectrophotometer was calibrated with a white tile having the following values: L* = 93.5, a* = 1.0, b* = 0.8. Analyses were performed at three different points on both the crumb and the crust areas, and the results were averaged.

### 2.4. Bread Image Analyses

Images of three bread crumb slices were captured using a Sony Alpha 6100 mirrorless digital camera (Sony Corporation, Tokyo, Japan). The images were saved as JPG format at a resolution of 350 dpi in the red-green-blue (RGB) color space for further analysis. Image segmentation was conducted using the ImageJ freeware (National Institute of Health, Bethesda, MD, USA) 1.54 g program (http://imagej.net/ij/, accessed on 15 July 2025). Measurements were obtained in pixels and converted into centimeters by using known scale. Each image was cropped to a 5 cm × 5 cm field of view and then converted to 8-bit grayscale. Segmentation was performed by binarization of grayscale images into black-and-white images using the Otsu algorithms in ImageJ, followed by an erosion step to remove noise [35,36]. Porosity was determined by calculating the percentage of pores relative to the total measured area [37].

Finally, the “3D Object Counter” plugin was used to quantify the pore characteristics, while the “Interactive 3D Surface Plot” plugin was employed to create 3D surface plots of bread, visualizing the pore distribution. Three replicate tests were conducted at each sample, and the results were averaged.

### 2.5. Determination of Free Amino Acids (FAA) in Breads

The extraction of FAA from the bread was performed by magnetically stirring 1.0 g of the freeze-dried sample for 30 min at 25 °C with 5 mL of 0.1 M HCl. The sample was then centrifuged at 20,000× *g* at 4 °C for 10 min (Centrifuge 5430 R model, Eppendorf AG, Hamburg, Germany). The resulting supernatant was then filtered through a 0.2 μm syringe filter.

Amino acids analyses were performed on an Agilent 1260 Infinity III Prime LC system (Agilent Technologies Inc., Santa Clara, CA, USA), according to the described methods, with slight modifications [38,39]. Separation was performed using a Gemini^®^ C18 (150 × 4.6 mm; 3 μm) analytical column (Phenomenex, Torrance, CA, USA) protected with a C18 security guard cartridge (4 × 3.0 mm i.d.; Phenomenex), with the oven temperature set to 40 °C. The mobile phase A and B consisted of sodium acetate (25 mmol/L at pH 8.0), and acetonitrile and methanol (50/50, *v*/*v*), respectively. The gradient program was set as follows (time (min), %B): 0/0, 0.5/2, 20.0/57, 20.10/100, 23.60/100, 23.70/2, and 24/2, at a flow rate of 1.5 mL/min. The total run time was 24 min. Samples were automatically derivatized with o-phthalaldehyde (OPA) and 9-fluorenyl methyl chloroformate (FMOC) (Agilent part number 5061–3335 and 5061–3337; Agilent Technologies Inc., Santa Clara, CA, USA) using an automated vial sampler (model G7129C) following the injection program described in the application note [38]. Quantification was performed using fluorescence detector (model G7121B). OPA derivatives were detected at 240 nm (excitation) and 450 nm (emission) from 0 to 13.5 min, while FMOC derivatives were monitored from 13.5 min at 240 nm (excitation) and 315 nm (emission). The injection volume was 2.0 µL. Peak identification was conducted by comparing retention times with those of standard solutions of 23 amino acids diluted in water, prepared according to the method described in the application note [38]. Calibration curves were prepared at concentrations ranging from 45 to 225 pmol/μL. Quantification of amino acids was achieved using OpenLAB CDS 2 ChemStation Edition 3.7 (Agilent Technologies Inc., Santa Clara, CA, USA).

### 2.6. Total Phenolic Content

The total phenolic content (TPC) of the bread samples was evaluated using an iCubio i-Magic M9 analyzer (Origlia S.r.L, Cornaredo, Italy). Analyses were performed with the EnzytecTM Polyphenols kit (Cod. E2530) from R-Biopharm (R-Biopharm AG, Darmstadt, Germany). Conventional solid-liquid extraction of polyphenols and sample preparation were performed according to the manufacturer’s instructions, and as previously reported [40], with slight modifications: 20 g of freeze-dried bread sample were treated with 80 mL of an ethanol solution (70% *v*/*v*) and magnetically stirred at room temperature for 60 min. The dispersions were then centrifuged at 15,000× *g* for 10 min at 15 °C (Centrifuge 5430 R model, Eppendorf AG, Hamburg, Germany). Subsequently, 8 mL of the supernatant was diluted with distilled water to a final volume of 10 mL. A calibration curve of absorbance vs. concentration of Gallic acid (GA) standard, in the range 0.02–3.0 g/L, was used to quantify the TPC. Results were expressed as mg GA equivalents per 100 g on a dry-weight basis (mg GAE/100 g d.b.) of bread. Extractions and measurements were performed in duplicate.

### 2.7. Determination of Antioxidant Activity

#### 2.7.1. DPPH Radical Scavenging Activity

The DPPH radical scavenging activity of bread extracts was measured according to the method previously reported [41]. A solution of DPPH (0.10 mM, 1.4 mL) in 80% aqueous ethanol and 0.1 mL of bread extract was mixed and allowed to stand in the dark for 30 min. The absorbance was then measured at 517 nm. A Trolox calibration curve in the range of 5–200 mg L^−1^ (y = −0.004x + 1.0694; R^2^ = 0.9994) was used to calculate the radical scavenging activity. Results were expressed as mg of Trolox equivalents (TE) per 100 g on a dry-weight basis (mg TE/100 g d.b.) of bread. The extractions and measurements were performed in duplicate.

#### 2.7.2. ABTS Radical Cation Scavenging Activity

The radical scavenging activity of bread extracts against the ABTS radical cation was measured using previously reported methods with some modifications [41]. A sodium persulfate stock solution (6.89 × 10^−3^ M, 1.0 mL) was added to an ABTS stock solution (5.0 × 10^−4^ M, 99.0 mL) and stored in the dark for 16 h before use. Fifty microlitres of each diluted sample (one of ten) were mixed with 1.45 mL of the ABTS solution. After reacting in the dark at 37 °C for 10 min, the absorbance at 734 nm was measured.

A Trolox calibration curve in the range 25–400 mg L^−1^ (y = −0.0015x + 0.7315; R^2^ = 0.9991) was used to determine the ABTS radical cation scavenging activity. Results were expressed as mg of Trolox equivalents (TE) per 100 g on a dry-weight basis (mg TE/100 g d.b.) of bread. The extractions and measurements were performed in duplicate.

### 2.8. Sensory Acceptability and CATA Evaluation

Sensory evaluation was conducted with a panel of untrained consumers (N = 65), predominantly female (65%) and mainly middle-aged (45–64 years). Participants were regular bread consumers, ensuring their relevance for evaluating product acceptability. All subjects provided written informed consent, outlining study objectives and potential allergen risks.

The study employed a combined approach, integrating quantitative hedonic scores with qualitative CATA descriptors to correlate sensory perceptions with affective responses. The test was conducted under real consumption conditions [42,43] in a calm, controlled, and quiet environment. Assessors were seated separately and had no contact with each other during the evaluation. Although the test was not carried out in individual sensory booths, the experimental design, sample presentation order, and the randomization and balancing procedures were defined in line with the general methodological principles described in ISO 11136:2014 and ISO 6658:2017 [44,45].

Testing was performed under blind conditions using three-digit coded samples to eliminate bias from labeling or health-related expectations. To ensure uniformity, bread samples underwent a standardized pretreatment: loaves were sliced (discarding end-pieces), and central slices were placed in a domestic dehydrator (10 min at 30 °C) to remove residual ethanol from the modified-atmosphere packaging. Slices were then individually sealed in a transparent, food-grade resealable bag to prevent cross-contamination, moisture loss, and uncontrolled aroma volatilization. Samples were stored at room temperature and evaluated within the same day.

The descriptors were pre-selected by integrating methodological guidelines for CATA [46,47] with standardized bread lexicons [48]. This list was then refined through focus group sessions involving food technologists and researchers who are also regular bread consumers. This qualitative phase ensured the terms were representative of the products’ sensory variability and aligned with consumer language. During these sessions, the initial list was refined by eliminating redundant, ambiguous, or poorly understood terms, while retaining descriptors considered meaningful and clearly interpretable. The final list encompassed aroma, taste, and texture/mouthfeel attributes, as well as flavor-related notes (integrating retronasal aroma and taste perception) and oral sensations (e.g., astringency). This process resulted in a fixed list of 17 descriptors used consistently throughout the study to ensure methodological transparency and reproducibility. To minimize bias, the order of descriptors was randomized across participants.

A total of nine samples were evaluated over three independent sessions (three samples per session) at ambient temperature (22 °C). Following a balanced Latin square design to minimize order and carryover effects, samples were presented monadically. Between assessments, participants were required to rinse their mouths with water to prevent sensory fatigue and residual flavor interference.

Participants first evaluated each bread using a 9-point hedonic scale (from 1 = “dislike extremely” to 9 = “like extremely”) for appearance/color, odor/aroma, taste/flavor, texture/mouthfeel, and overall liking. Immediately following the hedonic rating, consumers completed a CATA questionnaire consisting of the 17 sensory descriptors.

Responses were collected as binary data (1 = selected, 0 = not selected) and analyzed based on selection frequencies to determine the sensory profile of each product [46,47,49].

### 2.9. Statistical Analysis

All statistical analyses were performed using XLSTAT Luminero 2025 (Addinsoft, Paris, France). Physicochemical data, including color parameters, porosity, FAA composition, TPC, and antioxidant activity, were expressed as mean values ± standard deviation and analyzed by one-way analysis of variance (ANOVA). When significant effects were observed, Tukey’s HSD post hoc test was applied to identify differences among bread samples (*p* < 0.05). Pearson’s correlation coefficient (*r*) was calculated to assess the relationship between TPC and antioxidant activity indices (DPPH and ABTS) (*p* < 0.05).

Consumer hedonic data collected on a 9-point liking scale were treated as ordinal, as commonly recommended for consumer sensory data [50,51]. Data normality was assessed using the Shapiro–Wilk test and, since distributions were non-normal (*p* < 0.05), non-parametric statistical methods were applied. Differences among bread samples for appearance, odor, taste, texture, and overall liking were evaluated using the Kruskal–Wallis test. When significant differences were detected (*p* < 0.05), pairwise comparisons were performed using the Dunn–Bonferroni post hoc test.

Relationships among the five hedonic attributes (appearance, odor, taste, texture, and overall liking) were explored by Principal Component Analysis (PCA) performed on the correlation matrix of mean liking scores. PCA was used to identify the main dimensions underlying consumer acceptance and the contribution of individual sensory dimensions [50].

Binary CATA data were analyzed using Cochran’s Q test to detect significant differences in citation frequencies among bread samples for each sensory descriptor (*p* < 0.05). When the overall test was significant, pairwise comparisons between samples were performed using McNemar’s test with Bonferroni correction.

Correspondence Analysis (CA) was applied to CATA citation frequencies to visualize relationships between bread samples and sensory descriptors [52]. The stability of sample configurations obtained from CA was assessed by bootstrap resampling. In addition, a Principal Coordinate Analysis (PCoA) biplot was generated from the CATA frequency matrix to further explore sample separation and attribute associations.

Hierarchical Cluster Analysis (HCA) was performed on CATA citation frequencies to group bread samples according to similarities in their sensory profiles. Clustering was conducted using Ward’s linkage method and Euclidean distance as the dissimilarity measure, and results were visualized using a dendrogram. Consumer segmentation was further explored by applying hierarchical clustering to mean-centered individual liking scores in order to identify groups of consumers with similar preference patterns.

Penalty analysis (mean drop/mean impact) was carried out on CATA data to quantify the effect of the presence or absence of each sensory descriptor on overall liking. Mean impact values were interpreted in relation to attribute citation frequency to identify positive and negative drivers of consumer acceptance.

Overall, the integrated use of consumer hedonic evaluation, CATA, and multivariate statistical techniques enabled a comprehensive interpretation of sensory perception by linking specific product attributes to overall liking. The combined application of CA and PCA proved effective in identifying the main drivers of preference, supporting product optimization and quality improvement strategies. While CATA provided an efficient consumer-based profiling tool, multivariate analyses facilitated the interpretation of complex datasets by relating descriptor citation frequencies to hedonic responses [53].

## 3. Results and Discussion

### 3.1. Colour Parameters

Color is a crucial parameter that significantly influences consumer preferences for specific types of bread [5,54]. While sensory assessment of color relies on visual perception, instrumental analysis is essential for accurate detection and measurement, as it provides a level of precision that exceeds the capabilities of the human eye [1].

The results of the color evaluation for both the crumb and crust of the different bread samples are summarized in Table 2.

Bread color is a complex outcome driven by both formulation and baking parameters. Crumb color is primarily determined by the type of flour and the presence of pigmented ingredients such as seeds, grits, and whole grains. In contrast, crust color is influenced by the interaction between formulation and baking conditions. While crust browning is mainly the result of Maillard reactions and caramelization, the overall color development may also be influenced or masked by natural pigments present in the ingredients [55].

Regarding crumb lightness (L*), the darkest crumbs were observed in breads containing whole-grain or dark cereal flours. The rye-based sample GB-B showed the lowest L* value (55.96 ± 2.42), followed by the whole-grain formulations GB-D (60.70 ± 0.01) and PB-F (61.90 ± 1.34). Their reduced lightness is consistent with the higher content of bran, phenolic pigments, and seeds, all of which contribute to a darker crumb matrix [56,57].

In contrast, the lightest crumbs were found in breads produced with refined flours. Samples PB-I, GB-C, GB-A, PB-E, and PB-H displayed the highest L* values (ranging from 74.60 ± 0.38 to 77.91 ± 2.29) and were not significantly different from each other. These formulations, all based on low-extraction soft or durum wheat flours, contain minimal bran and therefore exhibit a significant brighter crumb.

The highest crumb a* value (8.58 ± 0.45) was recorded in the GB-B sample formulated with rye flour, followed by the whole-wheat breads PB-F (5.25 ± 0.10) and GB-D (4.90 ± 0.23). This pronounced redness is primarily attributed to the natural presence of bran flavonoids and other phenolic compounds, together with Maillard reaction products formed during baking [58,59].

Regarding the b* parameter, elevated yellowness values were observed in breads containing ingredients naturally rich in carotenoid pigments, such as durum wheat semolina and toasted corn grits. Samples PB-H, GB-C, and PB-G showed high b* values (approximately 23), consistent with their carotenoid-rich formulations [60]. Furthermore, the combination of rye flour and dark seeds in GB-B resulted in the highest b* value (27.52 ± 0.37), giving this sample its characteristic dark yellow-brown crumb appearance.

The darkest crusts, characterized by low L* values (ranging from 37 to 40), were found in GB-D, GB-B, and PB-F breads. This pronounced darkness is explained by their high content of bran- and germ-rich flours, which provides both inherent pigmentation and abundant precursors that enhance surface browning [61]. Conversely, breads based on low extraction flours, such as soft wheat Type “0” (PB-E, PB-I) or common wheat (GB-A), tended to have lighter crusts, with L* values between 51 and 54. A similar lightness was also seen in PB-G and PB-H (L* = 49), whose low extraction-flour base counterbalances the color contribution of mixed cereal flakes. Although these flours contain minimal bran, several of these formulations include malted barley flour (GB-A, PB-G, PB-I), which promotes a uniform golden-brown color by providing simple sugars for caramelization without generating the intense browning typically associated with whole-grain doughs [62]. Overall, the crust a* and b* parameters were significantly higher than the corresponding crumb values across all samples, a result of the Maillard reaction and the formation of melanoidins on the bread surface [54]. Regarding the redness index (a*), sample GB-C exhibited the highest a* value (16.98 ± 0.93), although it was statistically comparable to samples GB-B, PB-E, and PB-G (*p >* 0.05). This indicates that toasted corn grits (in GB-C), as well as the presence of dextrose and malt extract (in GB-B), are equally effective in promoting red-brown pigment formation. Finally, samples GB-A, PB-E, PB-G, and PB-I exhibited both higher b* values than the whole grain breads (GB-B, GB-D, PB-F), characteristic of the golden-brown crust typical of low extraction flour formulations.

### 3.2. Bread Image Analyses

The digital crumb appearance, binarized images, and reconstructed 3D surface plots of bread samples, generated via Image J, are presented in Table 3.

Reconstructed 3D surface plots offer a superior visualization of the interconnected cell network and are considered more effective than 2D methods for characterizing the overall cellular structure of bread. These models are generated by mapping the color or grayscale intensity of the original digital image onto the vertical dimension (Z-axis), thereby establishing a direct correlation between pixel intensity and crumb topography.

In these plots, the deep valleys (lowest Z-values), rendered in the magenta-violet spectrum, correspond to regions of highest porosity and represent the internal void space. The green color represents intermediate Z-values, indicating transition areas between the pores and the cell walls. Conversely, the surrounding peaks, which appear in a red-yellow spectrum, represent the solid matrix, highlighting areas of maximum material concentration.

Breads made with low extraction flour (GB-A, PB-G, PB-E, PB-I) were characterized by a soft, open crumb structure, with their 3D plots clearly showing deep and pronounced valleys. In contrast, the whole grain and rye flours used in GB-B, GB-D, and PB-F breads resulted in a denser, lower volume crumb with smaller air cells likely due to fiber particles disrupting the gluten network, resulting in flatter 3D plots with minor depressions. In addition, the inclusion of special ingredients such as corn grits or seeds (in GB-C and PB-H) introduced additional structural irregularities into the durum semolina cell matrix.

**Figure 1:**
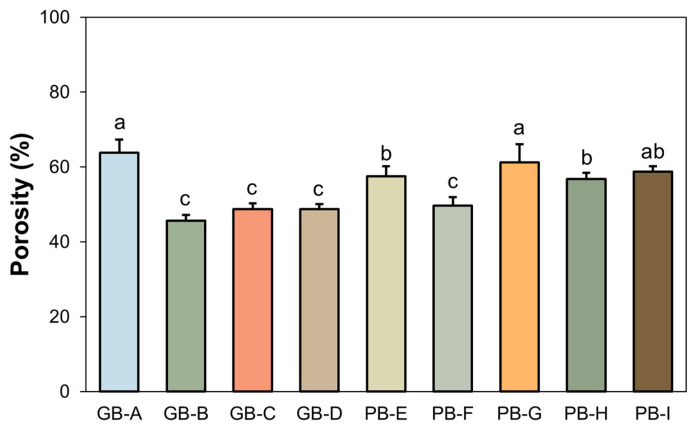
Porosity percentage of crumb breads. Values are mean ± standard deviation of triplicates. Different letters indicate a significant difference (*p* < 0.05).

The calculated percentage porosity of bread crumbs is reported in Figure 1.

According to the previous observations, GB-A, PB-G, and PB-I exhibited the highest porosity among all samples (63.8 ± 3.5%, 61.2 ± 4.8%, and 58.8 ± 1.3%, respectively), indicating a similarly well-developed gluten network. The matrix of GB-A is largely determined by high-quality common wheat flour with minimal disruptive inclusions (4% spelt and 1.5% semolina) [63]. Furthermore, the inclusion of dried sourdough likely contributes to the stabilization of the gluten network, leading to superior gas retention and a highly porous crumb [64]. Similarly, PB-G and PB-I benefited from refined soft-wheat bases and the addition of vital gluten, which helped counteract the disruptive influence of cereal flakes (in PB-G) and the combination of seeds and soy grits (in PB-I), thereby maintaining high porosity. Notably, PB-I was not significantly different from PB-E and PB-H, reflecting its intermediate-to-high porosity within the dataset.

Samples PB-E and PB-H showed porosity values ranging from 56% to 58%. These breads, although primarily formulated with low extraction flours, were slightly less porous than GB-A and PB-G due to differences in gluten quality and the presence of minor disruptive inclusions. PB-E achieved a relatively open structure through its high soft-wheat content, while PB-H relied on added gluten to compensate for the lower viscoelasticity of durum semolina.

The lowest porosity values (45–50%) were recorded for GB-B, GB-C, GB-D, and PB-F. These samples contain ingredients known to interfere with gluten development or gas entrapment. GB-B consisted of a high rye flour content (13%), whose pentosans are known to inhibit gluten formation [65], combined with a high loading of seeds. In GB-D and PB-F, the high whole-wheat content introduces bran and germ particles that physically disrupt the protein matrix. Finally, GB-C exhibited reduced porosity due to the combined effect of durum semolina, which forms a weaker network than common wheat, and disruptive inclusions such as corn grits and sunflower seeds.

### 3.3. FAA Quantification

**Figure 2:**
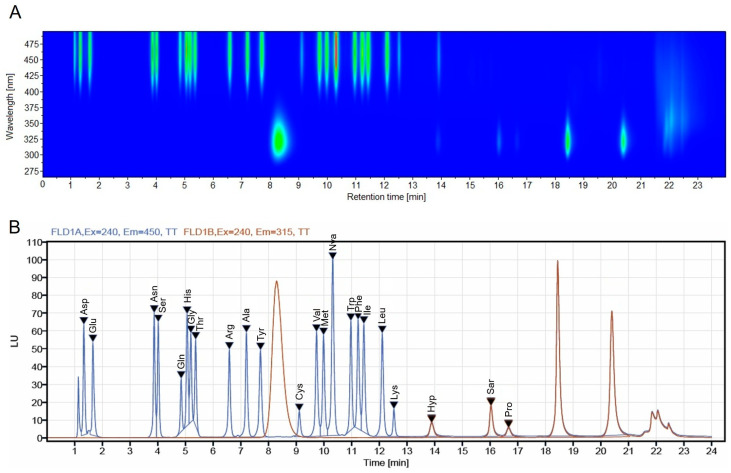
HPLC fluorescence Emission-Time-Maps at Excitation wavelength of 240 nm (**A**) and the extracted chromatogram of amino acid standard solution (**B**).

For the quantification of FAA following derivatization, emission wavelengths were optimized by online scanning at an excitation of 240 nm. The results, presented in a two-dimensional isoabsorbance plot (Figure 2A), display the peak intensity, wavelength, and retention time as a contour map.

The spectral information is presented as a series of isoabsorptive, concentric lines within the wavelength and time plane. These lines correspond to the retention peaks of the amino acids (showed in Figure 2B) and allow for a comprehensive visual inspection of all spectral data. Red and yellow regions indicate high and medium absorbance, respectively, while the blue region represent low absorbance.

Based on the isoabsorbance plots, the optimal maximum emission wavelengths were set at 450 nm for OPA-derivatized amino acids (corresponding to signals concentrated at higher wavelengths) and 315 nm for FMOC-derivatized amino acids. These two wavelengths were consequently selected for the fluorescence detection of the respective derivatized FAA.

The FAA composition of the bread samples is presented in Table 4.

Twenty-one amino acids, including seven essential amino acids (EAAs), were detected in nearly all samples. The highest Total AA values were found in samples where naturally protein-rich flour bases were combined with protein-boosting additives.

GB-B exhibited the highest concentration (94.4 ± 1.7 mg/100 g d.b.), which can be attributed to a significant stacking effect. This formulation includes added wheat gluten alongside a high percentage of common wheat flour and rye flour (13.1%). Notably, it is further enriched with three types of high-protein seeds (sunflower, sesame, flax), resulting in an exceptionally protein-dense profile [66]

PB-H also showed an extremely high Total AA value (88.0 ± 0.9 mg/100 g d.b.), driven by its primary ingredient, re-milled durum wheat semolina (68.2%) This is characterized by a naturally high protein content, and the synergistic effect of this base combined with supplemental wheat gluten leads to a robust concentration of total amino acids [67].

Regarding the PB-I sample, although it is based on soft wheat flour, its high total AA content (85.6 ± 6.1 mg/100 g d.b.) stems from the combined effect of added wheat gluten and a substantial inclusion of high-protein seeds and legumes (5% flax seeds, 4.3% soy grits, and 3.3% sunflower seeds). The protein contribution from these non-wheat sources, particularly the soy grits, significantly enhances the overall amino acid profile by providing a more diverse protein matrix [33].

Conversely, the lowest Total AA concentrations were observed for PB-E (44.2 ± 1.2 mg/100 g d.b.) and PB-G (45.2 ± 0.7 mg/100 g d.b.). The low value for PB-E is explained by its composition: the primary ingredient is soft wheat flour Type “0” (70.6%), which has an intrinsically lower protein content than whole wheat or durum semolina. Crucially, this bread contains no supplemental wheat gluten, protein-rich seeds, or soy grits. The low Total AA content for PB-G is comparable to PB-E, despite the inclusion of protein boosters (soy grits and wheat gluten). This suggests that the total flour to cereal ratio may be lower, or that the overall protein concentration in the final product is diluted by other non-proteinaceous ingredients. Specifically, its low Pro (1.0 ± 0.3 mg/100 g d.b.) and Glu (8.7 ± 0.4 mg/100 g d.b.) values, the primary AA in gluten, indicate a substantially lower effective amount of wheat protein in the finished bread compared to high-AA samples like PB-H or GB-B.

PB-F exhibited a high Total AA content (70.6 ± 0.5 mg/100 g d.b.) relative to the white bread (PB-E), yet lower than the most enriched samples. This is primarily driven by the high percentage of whole wheat flour (66.6%) and the inclusion of wheat gluten. Whole wheat flour incorporates the bran and germ, which are naturally richer in protein than low extraction flours, providing a higher baseline of amino acids that is further bolstered by the added gluten.

Since Glu and Pro are the predominant amino acids in gluten, their concentration serves as a reliable indicator of total wheat protein content. This is evidenced by the high Glu levels in PB-I and GB-B (14.8 ± 1.8 and 14.3 ± 0.3 mg/100 g d.b., respectively). The highest Pro values were recorded in PB-H (14.0 ± 0.5 mg/100 g d.b.), likely due to the re-milled durum semolina (68.2%), which is particularly abundant in proline-rich gluten fraction. This was followed by PB-I and GB-B, both showing values around 10 mg/100 g d.b.

Lys, widely recognized as the limiting essential amino acid (EAA) in cereal proteins, ranges from 1.1 to 1.9 mg/100 g d.b. It serves as a primary indicator of nutritional quality, with variability driven by the nature and proportion of non-wheat ingredients [68]. Low Lys content (ranging from 1.1 to 1.6 mg/100 g d.b.) was observed in samples dominated by wheat or semolina (PB-E, GB-D, PB-F, GB-A, PB-H, and PB-G). Specifically, reaching the lowest recorded values, PB-E consists primarily of refined soft wheat flour, which lacks the Lys-rich germ and bran. Additionally, the presence of added sugar likely facilitated the Maillard reaction during baking, leading to the thermal degradation of available Lys. Conversely, the addition of seeds or soy grits provided a significant Lys boost, as evidenced by the higher concentrations in GB-C, GB-B, and PB-I. Achieving the highest content (1.9 mg/100 g), PB-I benefits from the inclusion of soy grits, which are naturally rich in Lys. Similarly, samples GB-B and GB-C (1.8 mg/100 g) incorporate a diverse blend of sunflower, sesame, and flax seeds, offering a more robust amino acid profile. Finally, in GB-B, the use of rye flour further diversified the protein source, as rye typically possesses a more favorable EAA profile than refined common wheat.

Regarding other abundant AA, the highest Arg values were found in GB-B (15.1 ± 0.4 mg/100 g d.b.), PB-G (13.0 ± 0.1 mg/100 g d.b.), and PB-I (10.8 ± 1.3 mg/100 g d.b.). This pattern aligns with the high percentage of oilseeds (sunflower, sesame, and flax in GB-B) and soy grits (PB-G and PB-I), which are recognized as rich sources of Arg. Similarly, Asp levels peaked in GB-B (12.9 ± 1.4 mg/100 g d.b.) and PB-I (9.8 ± 2.4 mg/100 g d.b.), confirming the correlation between these amino acids and high-protein, non-wheat additions.

### 3.4. Total Phenolic Content and Antioxidant Activity

TPC and antioxidant activity of the selected breads were evaluated to investigate how functional ingredients, such as whole-grain flours, oilseeds, and sourdough, contribute to enhancing nutritional properties and promoting natural preservation. These ingredients act as effective clean-label alternatives to synthetic additives by extending shelf-life and protecting the product from lipid oxidation, while simultaneously improving the overall nutraceutical profile.

**Figure 3:**
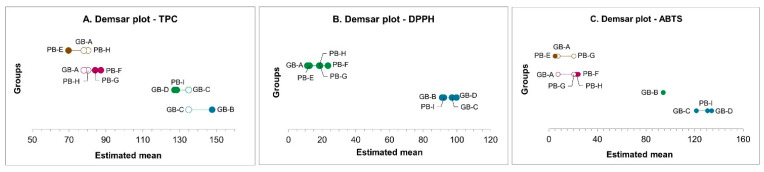
Demsar plots showing the significant difference of Total polyphenol content (**A**), DPPH (**B**), and ABTS (**C**) radical scavenging activity of bread samples (n = 2). Estimated mean were expressed as mg GAE/100 g d.b. for TPC, and as mg TE/100 g d.b. for DPPH and ABTS, respectively. Samples connected by a horizontal line are not statistically significantly different from each other at the chosen significance level (*p* < 0.05). Groups that are not connected are statistically different.

As shown in Figure 3A, bread samples PB-E, GB-A, PB-H, PB-F, and PB-G exhibited consistently low total phenolic content, ranging from 70 for PB-E to 87 mg GAE/100 g d.b. for PB-F.

This low TPC is directly attributed to the use of low extraction flours (such as Type “0” or re-milled semolina) as the primary ingredient, accounting for 63% to 71% of these formulations. The industrial milling process removes the bran and germ, which serve as the grain’s main repository of polyphenols [69].

Conversely, samples GB-D, PB-I, GB-C, and GB-B showed significantly (*p*  <  0.05) higher TPC, ranging from 128 (GB-D and PB-I) to 148 mg GAE/100 g d.b. found in the GB-B sample. This increase is linked to the substitution of low extraction flour with substantial amounts of phenolic- and lignan-rich ingredients. Specifically, these include whole grains (up to 56.2% in GB-D), oilseeds (sunflower, sesame, and flax), and functional flours such as rye (13.1% in GB-B). Several studies have documented that these components, particularly whole grain [70] and rye flour [71], which are rich sources of ferulic acid and bound phenolics, alongside lignan rich oilseeds, significantly enhance the overall antioxidant capacity of bread [72]. This evidence suggests that the variety and proportion of these functional ingredients are the primary drivers of the final TPC.

The antioxidant activity of bread samples was evaluated using the DPPH and ABTS assays, with results presented in Figure 3B and Figure 3C, respectively. The data separate the samples into two statistically distinct clusters based on their DPPH radical scavenging activity. The first cluster, comprising GB-A, PB-E, PB-H, PB-F, and PB-G, showed significantly lower values, ranging from 11.23 ± 1.23 to 23.51 ± 0.24 mg TE/100 g d.b. for GB-A and PB-F, respectively. On the other hand, the second cluster, including GB-B, PB-I, GB-D, GB-C, exhibited a significant increase in activity, with values ranging from 91 to 100 mg TE/100 g d.b.

Regarding the ABTS scavenging effect, the highest values (ranging from 94 to 134 mg TE/100 g d.b.) were obtained for the group including PB-I, GB-C, and GB-D, which showed a significant difference (*p*  <  0.05) compared to samples GB-A, PB-E, PB-H, PB-F, and PB-G (ranging from 5 to 24 mg TE/100 g d.b. for PB-E and PB-F, respectively). Sample GB-B (94.10 ± 4.48 mg TE/100 g d.b.) was clustered separately, indicating a scavenging activity that, while statistically lower than the top three samples, remained significantly higher than the second cluster.

**Figure 4:**
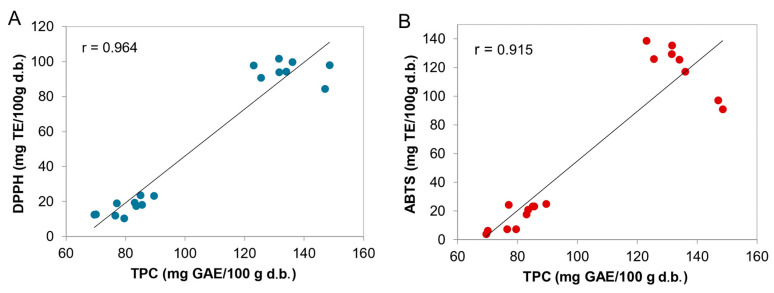
Correlation analysis between DPPH (**A**) and ABTS (**B**) scavenging activity and TPC, and Pearson correlation coefficient.

As illustrated in Figure 4, Pearson correlation analysis revealed a strong and significant positive correlation (*p* ≤ 0.05) between TPC and both DPPH (*r* = 0.964, Figure 4A) and ABTS (*r* = 0.915, Figure 4B) radical scavenging activity.

These findings confirm that phenolic compounds are the primary contributors to the antioxidant activities of the studied breads. In recent years, the incorporation of polyphenol-rich natural ingredients into bread formulations, aimed at improving the nutritional profile, health properties, and overall quality, has emerged as a compelling strategy to attract health-conscious consumers [73]. As is well established, the high nutritional value of phenolic compounds stems from their antioxidant, anti-inflammatory, and other health-promoting properties, which help protect against chronic diseases such as cancer, neurodegenerative, and cardiovascular disorders [74,75].

Additionally, these compounds enhance food quality by extending shelf life and preventing lipid oxidation, which mitigates spoilage and ensures chemical stability while providing natural color and flavor [76,77]. Our results highlight a clear positive correlation between the diversity of functional ingredients and antioxidant levels, aligning with the growing “Clean Label” trend in the bakery industry. This confirms that the strategic selection of raw materials directly impacts the chemical stability of the bread, protecting the final product from oxidative degradation.

Even within the “commercial sliced bread” category, there is a distinct technological shift toward formulations that replace synthetic additives, such as calcium propionate or sorbates, with functional components like seeds and ancient grains. By doing so, manufacturers achieve a dual benefit: natural preservation and an enhanced nutritional profile [13]. Ultimately, these findings demonstrate that modern bakery formulations are increasingly transitioning from synthetic chemistry toward functional, plant-based ingredients.

### 3.5. Sensory Acceptability and CATA Evaluation

**Figure 5:**
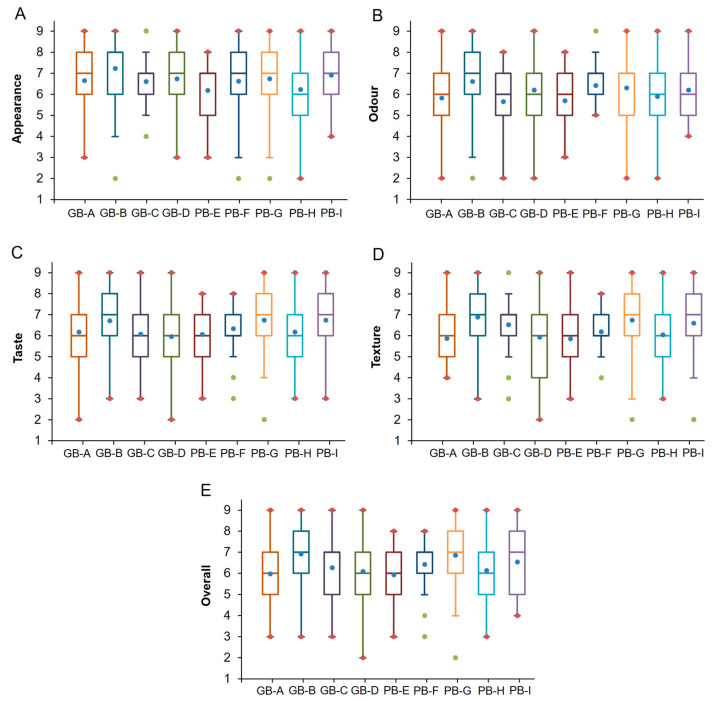
Boxplots of consumer liking scores for the nine bread samples across the four sensory attributes: appearance (**A**), odor (**B**), taste (**C**), texture (**D**), and overall (**E**). For each attribute, boxplots show the median, interquartile range (IQR), whiskers representing data dispersion, and outliers. Blue dots indicate the mean values. These plots provide a comparative overview of product acceptance and variability across samples for all sensory dimensions evaluated (n = 65).

As reported in Figure 5, the consumer hedonic evaluation of the nine bread samples revealed clear and systematic differences, demonstrating that the panel was highly effective in distinguishing the products based on all sensory dimensions: appearance (*p* = 0.001), odor (*p* < 0.0001), taste (*p* = 0.002), texture (*p* < 0.0001), and overall liking (*p* < 0.0001). These findings are consistent with previous studies highlighting the high discriminative power of hedonic ratings for cereal-based products [46,47].

Regarding appearance (Figure 5A), although median scores for most samples fell within a narrow range (6–7), the individual treatments showed distinct distributions. Samples PB-I, PB-G, and GB-B (alongside PB-F) were identified as the most visually appealing, characterized by higher quartiles and more concentrated positive ratings. Conversely, samples PB-E and PB-H exhibited greater dispersion and several low outliers, indicating higher variability in perceived visual quality among consumers, as previously observed [78].

Odor liking showed the widest variability among attributes (Figure 5B). While PB-I, PB-G, and PB-F achieved the highest medians, samples such as PB-H, PB-G, GB-C, and GB-A displayed low outliers (scores 2–3). This suggests that specific notes, potentially bran-like, earthy, or dried-fruit aromas, were disliked by a segment of consumers (Figure 5C).

Taste emerged as the most discriminating attribute (Figure 5C). PB-I and PB-G achieved the highest median scores (≥7), indicating highly appreciated flavor profiles. Samples with lower taste acceptance (PB-F, PB-E, and GB-A) were associated with bitter or bran-like notes, which are known to reduce consumer acceptance in cereal products [79,80].

Texture medians ranged between 6 and 7 (Figure 5D). PB-I, PB-G, and GB-C scored highest, while PB-E and GB-A were penalized for perceived dryness or excessive firmness.

The CATA results indicated that the descriptor ‘soft’ was frequently associated with samples showing higher texture liking, reflecting the complex and dynamic nature of texture perception during oral processing, which integrates mastication, oral lubrication, and saliva–food interactions, and cannot be fully described by instrumental static texture measurements alone.

Overall, liking varied significantly, with medians ranging from 6.0 to 7.0 (Figure 5E). PB-I, PB-G, and GB-C were the most preferred formulations, whereas PB-H, PB-E, GB-D, GB-B, and GB-A showed slightly lower acceptance. GB-D exhibited the most heterogeneous responses, indicating a lack of consensus among consumers. Pearson correlations confirmed that taste (*r* = 0.876) and texture (*r* = 0.844) were the strongest drivers of overall liking, consistent with findings in other baked goods [81], followed by odor (*r* = 0.653) and appearance (*r* = 0.614).

In summary, consumers consistently favored breads with balanced taste and texture profiles, particularly PB-I, PB-G, and GB-C, confirming that flavor and mouthfeel are primary drivers of acceptance. Formulations containing specific functional ingredients (e.g., PB-E, GB-A) face the technological challenge of masking bitter or earthy notes that affect sensory acceptance.

**Figure 6:**
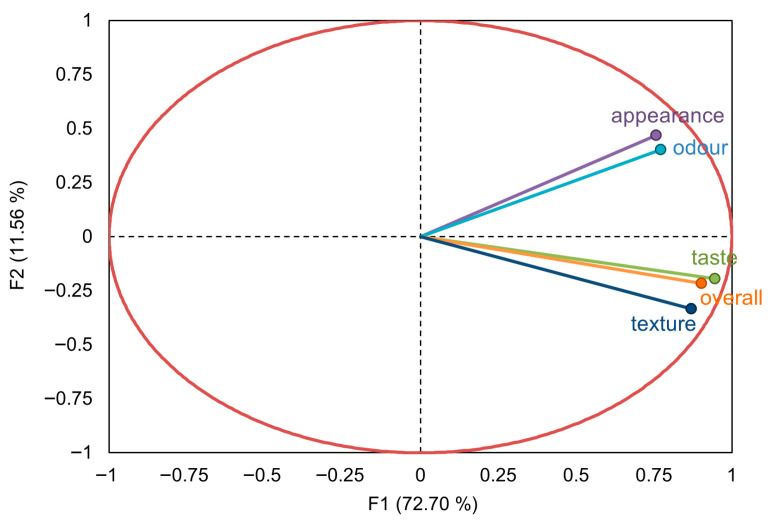
PCA loading plot for the five hedonic attributes (appearance, odor, taste, texture, and overall liking).

The relationships among the five hedonic attributes were further examined using PCA, as illustrated in the loading plot (Figure 6).

The two-dimensional PCA accounted for 84.26% of the total variance, providing a clear and representative overview of the data structure. The first principal component (F1), which alone explained 72.70% of the variance, emerged as the axis of general product acceptance. All attributes—appearance, odor, taste, texture, and overall liking—loaded strongly and positively on F1, confirming their interdependence and the high correlations typically observed among hedonic variables [41]. The close alignment of the overall liking, taste, and texture vectors indicates strong collinearity, suggesting that taste and texture are the primary drivers of consumer acceptance, with taste being the most influential factor.

The second principal component (F2), accounting for 11.56% of the variance, appears to capture an expectation–experience dimension. Appearance and odor liking loaded positively on F2 and clustered together, reflecting their role in the pre-consumption phase, where consumers form expectations about product quality based on initial sensory cues [78,82].

In contrast, taste and texture liking showed minimal variation along F2 and loaded primarily on F1. This indicates their dominance in the consumption or performance phase, where the actual sensory experience either confirms or contradicts prior expectations [83]. Collectively, the PCA confirms that while appearance and odor influence early expectations, taste and texture are the definitive contributors to overall liking. This two-phase interpretation, expectation versus confirmation, provides valuable insight for optimizing the sensory profiles of functional bakery products.

**Figure 7:**
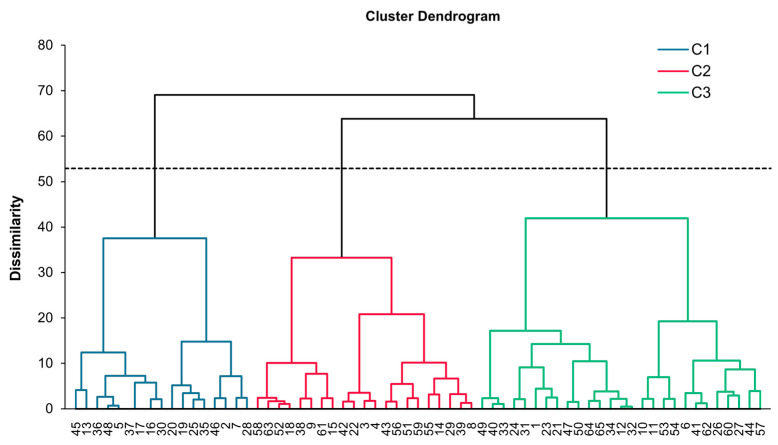
Hierarchical cluster analysis (HCA) dendrogram based on consumer liking patterns across the nine bread samples. Clustering was performed using Ward’s method and Euclidean distance as dissimilarity measure. The horizontal dashed line indicates the cut-off used to define three clusters (C1–C3). The numbers at the base of the dendrogram indicate the total number of consumers belonging to each identified cluster.

The Hierarchical Cluster Analysis (HCA) (Figure 7), based on consumer CATA frequencies, successfully partitioned the nine bread samples into three distinct groups (C1, C2, and C3).

The cut-off level (indicated by the dashed line) yielded three stable clusters: C1 grouped samples associated with less favorable attributes, such as adhesive texture, dryness, and intense cereal-related notes; C3 comprised products consistently linked to positive descriptors, including softness, easy of chew, sweet taste and umami; while C2 included samples with intermediate sensory profiles, suggesting a partial overlap between C1 and C3 clusters.

The dendrogram structure revealed the greatest dissimilarity between C1 and C3, confirming their divergence in perceived texture and flavor. Overall, the HCA demonstrated that consumer sensory perception effectively categorized the products into coherent groups, highlighting the strong discriminative power of the CATA descriptors.

**Figure 8:**
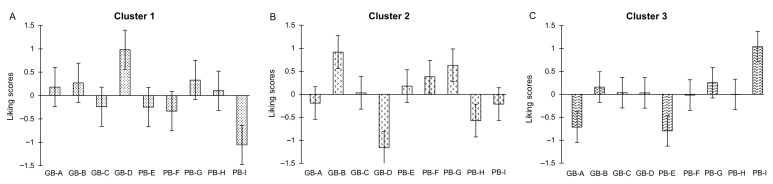
Liking scores for the bread samples in cluster 1 (**A**), cluster 2 (**B**), and cluster 3 (**C**). Mean centered liking scores (±SE) for the nine bread samples across the three consumer clusters identified through hierarchical clustering. Each subplot illustrates the relative preference patterns within each segment after centering individual ratings around the overall mean.

Beyond product grouping, hierarchical clustering also identified three distinct consumer segments (Clusters 1, 2, and 3), revealing pronounced heterogeneity in preference structures (Figure 8).

Cluster 1 showed a general negative orientation, particularly toward PB-I, which received most significant negative deviations (lowest centered scores, approximately −1.0). Conversely, GB-D was positively evaluated, suggesting that its sensory profile aligned well with this group’s expectations. Other GB-type samples were moderately accepted, though without prominent preference peaks.

Cluster 2 exhibited a strong preference for GB-B, which achieved the highest positive deviation. In contrast, GB-D was heavily penalized, indicating that its specific sensory characteristics were particularly disliked by this segment. Samples PB-G and PB-F showed mild positive deviations, reflecting partial acceptance.

Cluster 3 displayed a strong positive response to PB-I, the sample most strongly rejected by Cluster 1, while most other breads were rated near the mean. PB-E and GB-A received negative deviations, confirming their limited acceptance across consumer segments.

These results highlight substantial heterogeneity in consumer preferences. PB-I and GB-D emerged as the most polarizing samples, whereas GB-B performed consistently well in Clusters 2 and 3, but not in Cluster 1. Such findings underscore the necessity of product optimization strategies tailored to distinct consumer segments, rather than relying solely on aggregated liking scores.

As reported in Table 5, the analysis of the CATA data, supported by McNemar’s and Cochran’s tests, revealed a clear, descriptor-specific pattern of sensory differences among the nine bread samples. Texture and mouthfeel descriptors emerged as the strongest discriminators, with “dry” and “adhesive” showing highly significant variation (*p* < 0.0001).

This finding is particularly relevant for functional bakery products, where health benefits alone rarely compensate for poor sensory quality. The CATA method proved effective in capturing how consumers perceive the complex interplay between formulation, structure, and final product characteristics.

Negative texture attributes such as “dryness” and “cardboard texture” were concentrated in the lower-liked samples. GB-D showed the highest citation frequencies for “dry” (0.631) and “cardboard texture” (0.492), indicating a firmer, less cohesive, and undesirable texture. This aligns with its high whole-grain content (56.2%) and elevated fiber level (8.4%), both of which are known to increase water competition and reduce perceived moistness. GB-A also showed elevated dryness (0.554) and cardboard texture (0.277), which may be associated with rapid starch retrogradation typical of highly milled, low-extraction wheat flours, potentially further influenced by the protein matrix of spelt and semolina.

In contrast, PB-G exhibited the lowest dryness score (0.169), indicating a markedly superior mouthfeel in terms of perceived hydration.

Regarding “adhesive” texture, PB-H (0.369) showed the highest frequency and differed significantly from several other samples. This stickiness may be linked to the high total amino acid content and the robust gluten network characteristic of durum-semolina-rich formulations.

Aroma and flavor attributes also contributed substantially to sample discrimination (*p* < 0.0001). Bran-related descriptors, such as bran and legume flavor, and mushroom, were strongly associated with whole-grain formulations. “Bran flavor” reached its peak in GB-D (0.877) and PB-F (0.846), both rich in whole grains and seeds. In contrast, PB-H, PB-E, and GB-A were among the least associated with these notes, reflecting their low extraction flour bases.

The distribution of desirable flavor attributes, such as “umami” and “sweet”, was significantly non-uniform (*p* = 0.001 for umami; *p* < 0.0001 for sweet). GB-B showed the highest frequency for “umami taste” (0.677), followed by PB-G and GB-C (both 0.631). This is consistent with the presence of protein-rich ingredients, such as seeds and soy derivatives, which provide free Glu and Asp key precursors of umami perception. “Sweet taste” was most frequently associated with PB-H, PB-G, PB-E, and GB-A, largely driven by added sugars and, in the case of PB-H, by sweet-tasting amino acids such as Ala and Gly [84]. Conversely, GB-D consistently fell on the low end for both sweet and umami notes, reinforcing its distinct and generally less-liked flavor profile.

Negative flavor attributes also showed significant differences. GB-B was the only sample strongly associated with “bitter taste” (0.246), possibly due to seed-derived compounds or intensified proteolysis during the bread making process. Other breads (e.g., PB-H, PB-E, PB-I) showed minimal or near-zero frequencies, indicating an uneven distribution of off-flavors across the product set.

Finally, several attributes were perceived similarly across all samples, suggesting that they are either inherent to the product category or lacked sufficient variation to discriminate among samples. “Softness” (*p* = 0.062), “easy to chew” (*p* = 0.093), and “astringent” (*p* = 0.082) yielded non-significant results, indicating that despite their importance for overall liking, their intensity did not differ statistically among the samples. “Acid taste” was also clearly non-significant (*p* = 0.848), suggesting a uniform profile regarding acidity across the set.

Notably, descriptors like “mushroom” were spontaneously generated by consumers to describe earthy or umami-like nuances typical of fermented, bran-rich matrices. Although mushrooms were not an ingredient, including such consumer-derived terms reflects their intuitive sensory language. While capturing authentic perception, this approach may involve metaphorical associations typical of consumer-led CATA lexicons.

**Figure 9:**
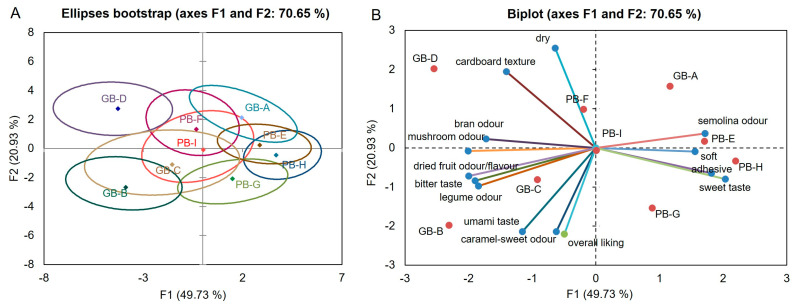
Bootstrap validation of sample positions obtained from Correspondence Analysis (CA) on CATA frequency data (**A**). Principal Coordinate Analysis (PCoA) biplot based on CATA citation frequencies for the nine bread samples (**B**). The first two dimensions (F1 = 49.73%, F2 = 20.93%) explain 70.65% of total variance. The plot shows the relationships between samples, sensory attributes, and overall liking, indicating which descriptors are most closely associated with consumer acceptance.

The bootstrap ellipses in Figure 9A illustrate the stability and consistency of the sensory profiles obtained from Correspondence Analysis (CA) on CATA frequency data.

Compact ellipses, such as those of PB-I, PB-G, and GB-B, indicate high positional stability and reflect consistent consumer citation patterns. Conversely, wider ellipses (e.g., GB-D, GB-A, PB-F, PB-E) suggest greater variability or heterogeneous perception among assessors. The clear separation between ellipses confirms that consumers perceived distinct sensory differences, whereas overlap would indicate sensory similarity. Overall, the CA map supports robust sample discrimination: highly liked products cluster compactly in the favorable region, while less-preferred products appear more dispersed.

Specifically, PB-I, PB-G, and GB-B cluster in the central/lower-right area (positive F1), associated with favorable descriptors such as soft texture, sweet taste, umami, and pleasant aromatic notes previously identified as hedonic drivers. The intermediate group (PB-F and PB-E), located in the upper-central region, shows moderate variability and is linked to intermediate intensities of cereal and semolina notes. Finally, GB-D, GB-C, and GB-A form a distinct group in the left or upper-left region; these samples exhibit broad ellipses, indicating higher inconsistency, and are associated with negative descriptors such as dry, adhesive, bran odor, and bitter taste.

The PCoA biplot (Figure 9B), explaining 70.65% of the variance (F1 = 49.73%, F2 = 20.93%), reveals the primary drivers of sensory discrimination and their alignment with overall liking. Axis 1 (F1) represents the dominant contrast in odor and flavor quality. The positive side is associated with favorable descriptors such as sweet taste, semolina and cereal odor, and soft texture. Samples from the PB series (particularly PB-E, PB-H, and PB-G) and GB-A are clearly defined by these descriptors; the projection of overall liking in this same direction confirms their positive contribution to consumer acceptance. Specifically, GB-A is associated with a drier profile, while PB-G aligns closely with the overall liking and umami vectors. Conversely, the negative F1 region is linked to less desirable notes, which were negatively correlated with liking. Samples GB-B, GB-C, and GB-D are specifically characterized by these earthy and bitter notes, including mushroom, legume, and bran odors. Axis 2 (F2) provides additional discrimination based on texture, with dryness and cardboard texture aligned toward the upper portion of the plot, further distinguishing samples like GB-D (high cardboard texture) from the softer PB samples.

The combined interpretation of CA and PCoA indicates that odor-flavor quality, specifically the contrast between sweet/cereal notes and bitter/bran notes, is the primary axis of sensory differentiation, followed by texture-related descriptors (dryness, softness, adhesiveness).

These findings indicate that consumer acceptance is primarily driven by the positive flavor and mouthfeel characteristics found in the PB series, whereas the negative sensory notes and unfavorable texture associated with the GB-B, GB-C, and GB-D samples lead to lower liking scores.

**Figure 10:**
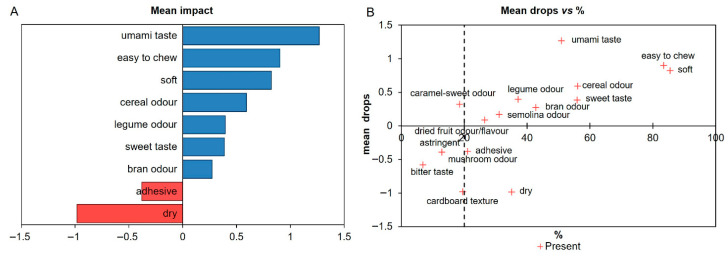
Mean impact plot derived from a penalty analysis based on CATA data (**A**). The graph shows the effect of the presence of each sensory attribute on overall liking. Bars to the right of zero indicate attributes associated with an increase in liking (positive penalties), whereas bars to the left indicate attributes associated with a decrease in liking (negative penalties). Mean drop vs. citation frequency (%) of CATA descriptors across samples (**B**).

Finally, the mean impact plot (Figure 10A) quantifies how the presence of each specific sensory attribute influences overall consumer liking.

Among the positive drivers, umami taste showed the strongest positive effect, marking it as a key enhancer of consumer acceptance. However, its citation frequency was moderate, implying a high benefit when present, though it is not as universally perceived as texture cues. Favorable mouthfeel attributes, specifically “easy to chew” and “softness”, were both highly frequent and strongly positive, identifying them as reliable, high-leverage targets for product optimization. Additional positive cues, such as cereal and legume odor and sweet taste, exerted smaller but beneficial effects.

In contrast, “dry” and “adhesive” textures exerted the most severe negative impacts on liking. The “dry” attribute showed a pronounced penalty (approximately −1.1), underscoring dryness as a critical defect. “Adhesive” texture was associated with a moderate negative impact (≈−0.4); while detrimental, its effect was less pronounced than that of dryness. These results clearly indicate that textural defects are the most penalizing factors for consumer acceptance, with dryness representing the primary texture-related risk.

Figure 10B displays the relationship between citation frequency (% Present) and hedonic impact (Mean Drops), offering insight into the primary drivers of preference and rejection. The upper-right quadrant with high frequency and positive impact includes “soft”, “easy to chew”, “umami taste”, and “sweet taste”. Among these, softness and easy to chew stand out due to their very high frequency combined with positive mean drops, confirming their role as core drivers of consumer preference. Umami remains a high-value enhancer even at moderate frequency. Strong negative impacts were observed for dryness and cardboard texture, which penalize liking substantially even at moderate frequencies. Low-frequency negative drivers, such as bitter taste and astringent, significantly decrease liking when present. Conversely, caramel-sweet odor showed a positive effect at low frequency, suggesting its potential as a niche, context-dependent enhancer. Attributes near a mean drop of ≈0, such as bran flavor (weakly positive) and cereal odor, exerted low net influence.

Integrating CA and PCoA with penalty analysis confirms that differences in product acceptance are primarily driven by flavor and mouthfeel perception. While appearance and odor contribute to discrimination, they are less influential in determining final liking.

Consistency across all analyses allowed for a clear grouping based on consumer preference. Samples PB-I, PB-G, and GB-B emerged as the most preferred, characterized by a balanced flavor, favorable texture, and appealing structure. In contrast, PB-H, PB-E, GB-A, and GB-D showed lower acceptance, consistent with the presence of negative descriptors such as dry, adhesive, bran flavor, or bitter taste. These findings reinforce that taste and mouthfeel are the strongest predictors of consumer liking, aligning with established literature on grain-based products. Consequently, product optimization should strategically focus on enhancing desirable textural and flavor attributes while mitigating negative textural and taste defects.

## 4. Conclusions

This study demonstrates that consumer perception of “clean-label” breads results from a complex interplay between composition, structural properties, and sensory attributes. Physicochemical analyses confirmed that formulations enriched with whole grains, oilseeds, or legume-derived ingredients exhibited superior nutritional profiles, characterized by higher levels of total phenolics, antioxidant activity, and free amino acids. However, these functional inclusions also introduced specific sensory notes and textural profiles that can challenge consumer acceptance.

Conversely, breads formulated with highly milled, low-extraction wheat flours displayed greater porosity and softer texture, which directly correlated with higher liking scores. The integration of CATA and penalty analysis identified softness, ease of chewing, sweetness, and umami as the primary drivers of consumer preference. The quantification of free amino acids provided the biochemical basis for these taste drivers, identifying them as core enhancers of overall acceptance.

In contrast, dryness, adhesiveness, bran-like flavors, and bitterness were confirmed as the main factors leading to product rejection.

The synthesis of physicochemical and sensory data highlights a critical technological challenge: the necessity to optimize texture and flavor when developing nutritionally enriched clean-label products. Specifically, the results emphasize that achieving high porosity and balancing the flavor impact of bioactive compounds are key to market success. These insights provide a strategic framework for manufacturers to design next-generation bakery products that successfully balance functional value with high consumer acceptability, meeting the demands of the modern health-conscious market without compromising the sensory experience.

Further studies are needed to investigate how innovative processing techniques or natural fermentation can mitigate negative attributes such as “dryness” and “cardboard texture” in high-fiber formulations, while also evaluating the impact of these clean-label strategies on the bread’s shelf-life and chemical stability.

## Figures and Tables

**Table 1:** Appearance of different bread slices and ingredients recorded from labels. * Percentages are expressed on the finished product.

Sample	Appearance	Ingredients
GB-A	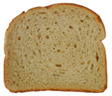	Common wheat flour type “0” (62.8%), water, spelt flour (wheat) (4%), extra virgin olive oil (3.3%), durum wheat semolina (1.5%), yeast, dried sourdough (wheat), salt, malted barley flour. Treated on the surface with ethyl alcohol.
GB-B	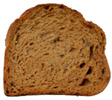	Common (soft) wheat flour type “0” (44.6%), water, rye flour (13.1%), sunflower seeds (4.0%), extra virgin olive oil (3.3%), wheat gluten, sesame seeds (2.0%), flax seeds (1.8%), dextrose (1.7%), yeast, dried rye sourdough (fermented rye flour, water), salt, barley malt extract. Treated on the surface with ethyl alcohol.
GB-C	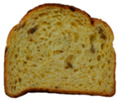	Durum wheat re-milled semolina (48.6%), water, toasted corn grits (8.0%), sunflower seeds (6.7%), sunflower oil (5.3%), yeast, wheat gluten, salt, malted barley, wheat fiber. Treated on the surface with ethyl alcohol.
GB-D	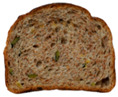	Whole wheat flour (56.2%), water, sunflower seeds (7.3%), sunflower oil (3.2%) *, yeast, wheat gluten, walnuts (1.8%), pumpkin seeds (1.4%), salt. Treated on the surface with ethyl alcohol.
PB-E	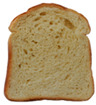	Soft wheat flour type “0” (70.6%), water, extra virgin olive oil (2.4%), yeast, salt, sugar, malted barley flour. Treated on the surface with ethyl alcohol.
PB-F	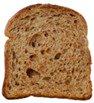	Whole wheat flour (66.6%), water, sunflower oil (5.8 %), wheat gluten, yeast, salt. Treated on the surface with ethyl alcohol.
PB-G	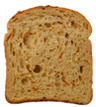	Soft wheat flour type “0” (56%), water, mixed cereal flakes and flours (6%) [oats (1.2%), barley (1.2%), spelt (wheat) (0.9%), durum wheat (0.6%), corn (0.6%), rice (0.6%), rye (0.6%), millet (0.3%)], soy grits (5.1%), sunflower oil (3.6%), yeast, wheat gluten, salt, malted barley flour, barley malt extract. Treated on the surface with ethyl alcohol.
PB-H	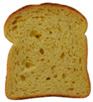	Durum wheat re-milled semolina (68.2%), water, extra virgin olive oil (2.9%), yeast, salt, sugar, wheat gluten, malted barley flour, soft wheat flour type “0”. Treated on the surface with ethyl alcohol.
PB-I	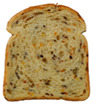	Soft wheat flour type “0” (55.5%), water, flax seeds (5%), soy grits (4.3%), sunflower seeds (3.3%), wheat gluten, yeast, extra virgin olive oil (1.2%), flaxseed oil (0.9%) (flax seeds, emulsifier, soy lecithin), salt, wheat fibre (0.6%), malted barley flour, vitamin E (0.03%). Treated on the surface with ethyl alcohol.

**Table 2:** Crumb and crust color analysis of bread samples expressed as L* (lightness), a* (red/green index), and b* (blue/yellow index) values.

Sample	Crumb Parameters			Crust Parameters		
	L*	a*	b*	L*	a*	b*
GB-A	75.05 ± 3.07 ^a^	0.90 ± 0.08 ^e^	15.51 ± 0.72 ^d^	54.08 ± 5.38 ^a^	13.00 ± 1.74 ^bc^	30.53 ± 0.20 ^a^
GB-B	55.96 ± 2.42 ^d^	8.58 ± 0.45 ^a^	27.52 ± 0.37 ^a^	37.62 ± 1.64 ^cd^	15.14 ± 0.42 ^ab^	20.69 ± 1.13 ^d^
GB-C	74.65 ± 1.43 ^a^	1.52 ± 0.15 ^d^	23.17 ± 0.88 ^b^	44.50 ± 2.75 ^bc^	16.98 ± 0.93 ^a^	27.79 ± 0.67 ^b^
GB-D	60.70 ± 0.01 ^cd^	4.90 ± 0.23 ^b^	15.09 ± 0.16 ^d^	37.07 ± 1.58 ^d^	12.45 ± 0.55 ^c^	18.42 ± 1.76 ^d^
PB-E	77.21 ± 1.52 ^a^	0.60 ± 0.13 ^f^	16.70 ± 0.30 ^d^	51.15 ± 0.80 ^ab^	14.71 ± 0.83 ^abc^	30.00 ± 0.42 ^a^
PB-F	61.90 ± 1.34 ^c^	5.25 ± 0.10 ^b^	19.01 ± 0.52 ^c^	40.26 ± 1.68 ^cd^	12.45 ± 0.19 ^c^	20.15 ± 0.01 ^d^
PB-G	69.02 ± 2.06 ^b^	3.62 ± 0.39 ^c^	22.67 ± 0.28 ^b^	49.64 ± 0.78 ^ab^	14.83 ± 0.42 ^ab^	30.31 ± 0.32 ^a^
PB-H	77.91 ± 2.29 ^a^	0.48 ± 0.16 ^f^	23.73 ± 0.59 ^b^	49.00 ± 2.98 ^ab^	12.93 ± 0.42 ^bc^	25.97 ± 0.76 ^c^
PB-I	74.60 ± 0.38 ^a^	1.26 ± 0.17 ^d^	16.84 ± 1.29 ^d^	53.71 ± 1.72 ^a^	13.22 ± 0.56 ^bc^	31.10 ± 1.34 ^a^

Values are mean ± standard deviation of triplicates. Within the same column, different letters indicate a significant difference (*p* < 0.05).

**Table 3:** Digital 8-bit grayscale images (50 × 50 mm crumb area) of bread samples, binary images thresholded using the Otsu algorithms, and reconstructed 3D surface plot images from the Image J.

Sample	Digital Images (50 × 50 mm Crumb Area) of Bread Samples	Binary Images Thresholded Using the Otsu ImageJ Algorithms	Reconstructed 3D Surface Plot Images
GB-A	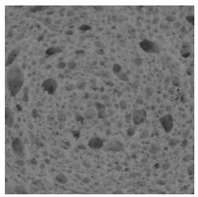	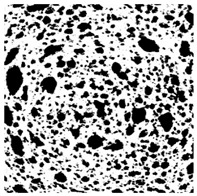	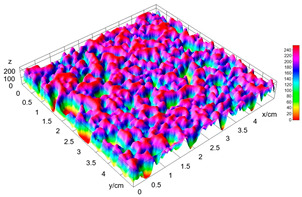
GB-B	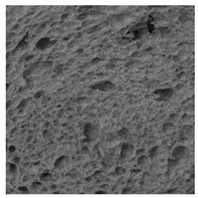	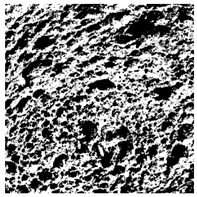	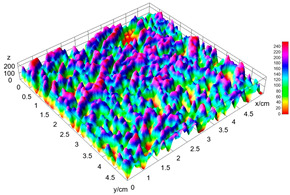
GB-C	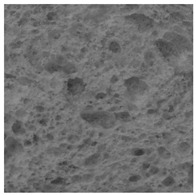	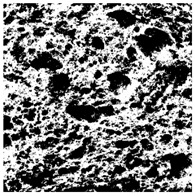	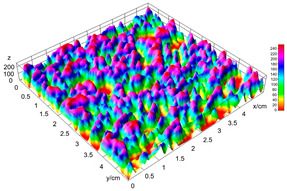
GB-D	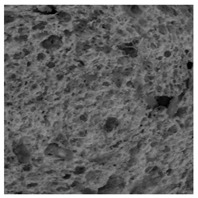	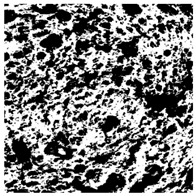	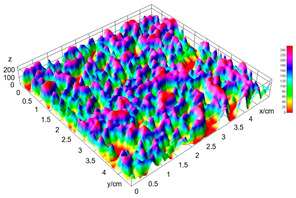
PB-E	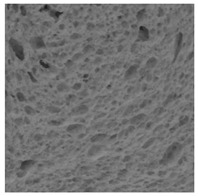	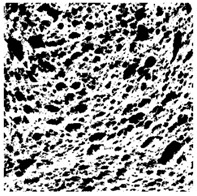	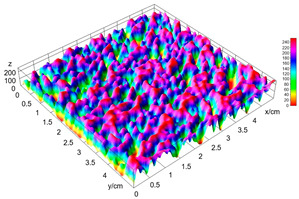
PB-F	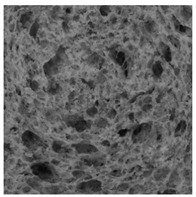	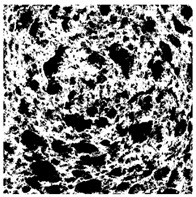	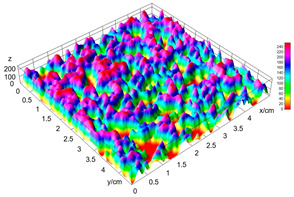
PB-G	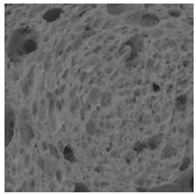	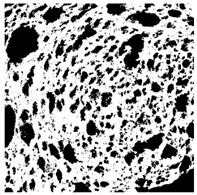	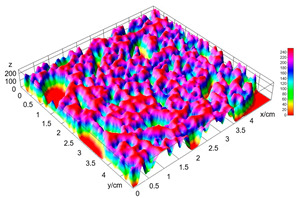
PB-H	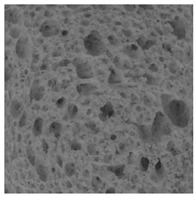	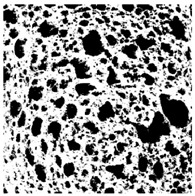	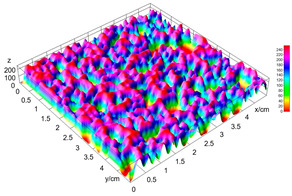
PB-I	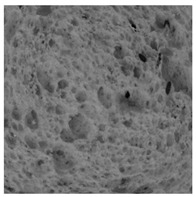	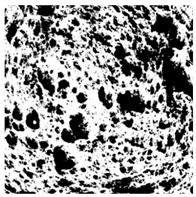	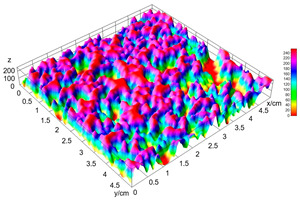

**Table 4:** Individual free amino acid content (mg/100 g d.b.) determined in different breads.

AA	GB-A	GB-B	GB-C	GB-D	PB-E	PB-F	PB-G	PB-H	PB-I
Asp	5.8 ± 1.55 ^d^	12.9 ± 1.40 ^a^	6.9 ± 2.15 ^cd^	5.8 ± 0.51 ^d^	7.1 ± 0.74 ^c^	9.1 ± 1.29 ^b^	3.3 ± 0.29 ^e^	7.3 ± 0.74 ^c^	9.8 ± 2.42 ^b^
Glu	9.5 ± 1.13 ^de^	14.3 ± 0.34 ^ab^	13.9 ± 2.25 ^abc^	9.9 ± 0.12 ^d^	8.8 ± 0.25 ^e^	10.1 ± 0.23 ^d^	8.7 ± 0.36 ^e^	12.3 ± 0.27 ^bc^	14.8 ± 1.78 ^a^
Asn	1.2 ± 0.09 ^e^	5.9 ± 0.19 ^a^	2.5 ± 0.13 ^c^	2.0 ± 0.06 ^d^	0.7 ± 0.01 ^f^	1.8 ± 0.09 ^de^	1.8 ± 0.09 ^de^	2.7 ± 0.12 ^c^	3.5 ± 0.01 ^b^
Ala	1.7 ± 0.10 ^e^	3.1 ± 0.57 ^bc^	2.5 ± 0.68 ^cd^	2.8 ± 0.01 ^cd^	1.4 ± 0.01 ^f^	3.6 ± 0.08 ^ab^	1.4 ± 0.17 ^f^	3.9 ± 0.04 ^a^	1.8 ± 0.31 ^e^
Arg	8.6 ± 0.17 ^d^	15.1 ± 0.43 ^a^	11.2 ± 1.25 ^c^	8.9 ± 0.10 ^d^	5.5 ± 0.06 ^e^	11.5 ± 0.38 ^c^	13.0 ± 0.08 ^b^	4.8 ± 0.60 ^e^	10.8 ± 1.28 ^c^
Gln	0.8 ± 0.08 ^d^	1.2 ± 0.05 ^bc^	1.2 ± 0.40 ^bc^	0.5 ± 0.03 ^f^	0.4 ± 0.04 ^g^	0.3 ± 0.01 ^h^	0.6 ± 0.01 ^e^	1.4 ± 0.04 ^b^	2.2 ± 0.16 ^a^
Gly	4.3 ± 0.61 ^bc^	4.3 ± 0.11 ^bc^	3.6 ± 0.01 ^d^	4.7 ± 0.29 ^ab^	3.3 ± 0.05 ^e^	4.2 ± 0.11 ^c^	3.0 ± 0.09 ^f^	4.8 ± 0.04 ^a^	1.6 ± 0.08 ^g^
Cys	2.7 ± 0.61 ^bc^	2.4 ± 0.21 ^cd^	2.5 ± 0.71 ^bc^	2.7 ± 0.02 ^bc^	2.2 ± 0.14 ^de^	2.8 ± 0.05 ^b^	1.9 ± 0.13 ^e^	2.9 ± 0.25 ^b^	3.9 ± 0.25 ^a^
Ser	1.6 ± 0.22 ^de^	2.1 ± 0.01 ^a^	1.5 ± 0.05 ^ef^	1.8 ± 0.11 ^bc^	1.3 ± 0.09 ^g^	1.4 ± 0.03 ^fg^	1.5 ± 0.12 ^ef^	1.7 ± 0.03 ^cd^	1.9 ± 0.11 ^b^
Tyr	4.3 ± 0.11 ^de^	4.7 ± 0.28 ^d^	3.5 ± 0.38 ^ef^	5.7 ± 0.15 ^c^	2.9 ± 0.40 ^f^	6.2 ± 0.10 ^bc^	6.8 ± 0.46 ^b^	5.8 ± 0.04 ^c^	7.7 ± 0.83 ^a^
His	0.9 ± 0.02 ^d^	1.0 ± 0.09 ^c^	0.9 ± 0.05 ^de^	0.9 ± 0.13 ^de^	0.8 ± 0.08 ^e^	0.8 ± 0.10 ^e^	1.3 ± 0.10 ^b^	0.8 ± 0.02 ^e^	1.4 ± 0.01 ^a^
Ile	0.2 ± 0.01 ^g^	1.3 ± 0.02 ^a^	0.8 ± 0.06 ^c^	0.8 ± 0.03 ^c^	0.3 ± 0.01 ^f^	0.6 ± 0.01 ^d^	0.5 ± 0.01 ^e^	0.5 ± 0.01 ^e^	0.9 ± 0.04 ^b^
Leu	0.7 ± 0.09 ^cd^	1.9 ± 0.01 ^a^	0.9 ± 0.12 ^bc^	0.9 ± 0.02 ^bc^	0.6 ± 0.01 ^d^	0.9 ± 0.02 ^bc^	0.9 ± 0.04 ^bc^	0.6 ± 0.13 ^d^	1.0 ± 0.03 ^b^
Lys	1.5 ± 0.21 ^cd^	1.8 ± 0.01 ^ab^	1.8 ± 0.64 ^abc^	1.4 ± 0.22 ^de^	1.1 ± 0.02 ^e^	1.6 ± 0.03 ^bc^	1.6 ± 0.10 ^bc^	1.6 ± 0.51 ^bc^	1.9 ± 0.06 ^a^
Met	1.4 ± 0.47 ^abc^	1.5 ± 0.25 ^ab^	1.3 ± 0.22 ^bc^	1.1 ± 0.02 ^de^	0.9 ± 0.02 ^e^	1.4 ± 0.05 ^ab^	1.2 ± 0.42 ^bcd^	1.5 ± 0.04 ^a^	1.2 ± 0.09 ^cd^
Phe	1.4 ± 0.24 ^b^	2.1 ± 0.05 ^a^	0.7 ± 0.03 ^d^	0.7 ± 0.07 ^d^	0.5 ± 0.01 ^f^	1.0 ± 0.02 ^c^	0.6 ± 0.03 ^e^	0.6 ± 0.01 ^e^	1.0 ± 0.06 ^c^
Thr	1.6 ± 0.24 ^cd^	2.2 ± 0.01 ^a^	0.4 ± 0.11 ^g^	1.6 ± 0.01 ^c^	1.3 ± 0.01 ^e^	1.6 ± 0.01 ^c^	1.2 ± 0.03 ^f^	1.9 ± 0.01 ^b^	1.6 ± 0.26 ^cd^
Trp	4.5 ± 0.60 ^d^	4.1 ± 0.01 ^de^	7.6 ± 1.03 ^ab^	6.3 ± 0.16 ^c^	3.8 ± 0.03 ^e^	8.3 ± 0.02 ^a^	2.8 ± 0.03 ^f^	8.4 ± 0.07 ^a^	6.5 ± 0.31 ^bc^
Val	1.5 ± 0.26 ^bc^	2.5 ± 0.11 ^a^	1.1 ± 0.19 ^cd^	1.0 ± 0.05 ^de^	0.7 ± 0.01 ^f^	1.6 ± 0.01 ^b^	0.7 ± 0.01 ^f^	1.1 ± 0.01 ^d^	1.3 ± 0.06 ^c^
Pro	8.8 ± 0.19 ^c^	10.1 ± 0.73 ^b^	3.7 ± 0.19 ^d^	4.1 ± 0.78 ^d^	0.8 ± 0.08 ^f^	1.7 ± 0.24 ^e^	1.0 ± 0.29 ^ef^	14.0 ± 0.51 ^a^	10.6 ± 0.98 ^b^
Total NEAA	49.1 ± 0.21 ^d^	76.2 ± 0.17 ^a^	52.9 ± 0.65 ^c^	49.0 ± 0.14 ^d^	34.3 ± 0.65 ^e^	52.8 ± 0.21 ^c^	34.6 ± 0.32 ^e^	70.1 ± 0.55 ^b^	68.9 ± 3.14 ^b^
Total EAA	14.3 ± 0.76 ^c^	18.2 ± 1.51 ^a^	16.6 ± 0.47 ^ab^	15.2 ± 0.20 ^bc^	9.9 ± 1.02 ^d^	17.9 ± 0.28 ^a^	10.6 ± 0.36 ^d^	17.9 ± 0.42 ^a^	16.7 ± 1.20 ^ab^
Total AA	63.4 ± 3.91 ^de^	94.4 ± 1.68 ^a^	67.8 ± 1.49 ^d^	64.2 ± 0.55 ^de^	44.2 ± 1.18 ^f^	70.6 ± 0.47 ^cd^	45.2 ± 0.69 ^f^	88.0 ± 0.90 ^b^	85.6 ± 6.09 ^bc^

Data are mean ± SD of individual sample twice analyzed. Within the same row, different letters indicate a significant difference (*p* < 0.05).

**Table 5:** Multiple pairwise comparisons between bread samples for each CATA descriptor using McNemar’s test with Bonferroni correction (N = 65 consumers). For each descriptor, superscript letters indicate significant differences between samples at α = 0.05 after adjustment for multiple testing. Samples sharing at least one letter are not significantly different, while samples with no letters in common differ significantly in citation frequency. The *p*-values reported in the rightmost column correspond to the overall Cochran’s Q test for each descriptor.

Attributes	GB-A	GB-B	GB-C	GB-D	PB-E	PB-F	PB-G	PB-H	PB-I	*p*-Values
Acid taste	0.077 ^a^	0.062 ^a^	0.092 ^a^	0.123 ^a^	0.062 ^a^	0.077 ^a^	0.123 ^a^	0.123 ^a^	0.092 ^a^	0.848
Adhesive	0.277 ^abc^	0.138 ^ab^	0.077 ^a^	0.077 ^a^	0.308 ^bc^	0.062 ^a^	0.354 ^bc^	0.369 ^c^	0.231 ^abc^	<0.0001
Astringent	0.092 ^a^	0.138 ^a^	0.215 ^a^	0.185 ^a^	0.154 ^a^	0.154 ^a^	0.077 ^a^	0.062 ^a^	0.077 ^a^	0.082
Bitter taste	0.015 ^a^	0.246 ^b^	0.062 ^a^	0.138 ^abc^	0.015 ^a^	0.046 ^a^	0.046 ^a^	0 ^a^	0.046 ^a^	<0.0001
Bran flavour	0.046 ^a^	0.692 ^de^	0.292 ^abc^	0.877 ^e^	0.154 ^ab^	0.846 ^e^	0.492 ^cd^	0.046 ^a^	0.400 ^bc^	<0.0001
Caramel-sweet	0.092 ^ab^	0.415 ^c^	0.292 ^bc^	0.108 ^ab^	0.108 ^ab^	0.062 ^a^	0.185 ^ab^	0.277 ^abc^	0.123 ^ab^	<0.0001
Cardboard texture	0.277 ^ab^	0.185 ^a^	0.169 ^a^	0.492 ^b^	0.108 ^a^	0.200 ^a^	0.077 ^a^	0.092 ^a^	0.154 ^a^	<0.0001
Cereal flavour	0.585 ^abcd^	0.677 ^cd^	0.462 ^abc^	0.646 ^bcd^	0.338 ^a^	0.815 ^d^	0.554 ^abcd^	0.400 ^ab^	0.569 ^abcd^	<0.0001
Dried fruit	0.077 ^ab^	0.523 ^c^	0.462 ^c^	0.523 ^c^	0.077 ^ab^	0.123 ^ab^	0.292 ^bc^	0.015 ^a^	0.292 ^bc^	<0.0001
Dry	0.554 ^cd^	0.231 ^ab^	0.262 ^ab^	0.631 ^d^	0.277 ^ab^	0.462 ^bcd^	0.169 ^a^	0.262 ^ab^	0.308 ^abc^	<0.0001
Easy to chew	0.785 ^a^	0.877 ^a^	0.815 ^a^	0.754 ^a^	0.877 ^a^	0.923 ^a^	0.892 ^a^	0.800 ^a^	0.785 ^a^	0.093
Legume flavour	0.138 ^a^	0.754 ^c^	0.508 ^bc^	0.615 ^bc^	0.031 ^a^	0.185 ^a^	0.477 ^b^	0.046 ^a^	0.585 ^bc^	<0.0001
Mushroom	0.138 ^abc^	0.277 ^c^	0.138 ^abc^	0.246 ^bc^	0.031 ^a^	0.092 ^ab^	0.108 ^abc^	0.015 ^a^	0.108 ^abc^	<0.0001
Semolina flavour	0.600 ^d^	0.123 ^a^	0.292 ^abc^	0.169 ^ab^	0.354 ^abcd^	0.185 ^ab^	0.400 ^bcd^	0.508 ^cd^	0.169 ^ab^	<0.0001
Soft	0.846 ^a^	0.785 ^a^	0.785 ^a^	0.785 ^a^	0.892 ^a^	0.923 ^a^	0.938 ^a^	0.877 ^a^	0.862 ^a^	0.062
Sweet taste	0.662 ^b^	0.462 ^ab^	0.508 ^ab^	0.277 ^a^	0.646 ^b^	0.538 ^ab^	0.692 ^b^	0.708 ^b^	0.538 ^ab^	<0.0001
Umami taste	0.400 ^ab^	0.677 ^b^	0.631 ^ab^	0.462 ^ab^	0.385 ^a^	0.462 ^ab^	0.631 ^ab^	0.431 ^ab^	0.508 ^ab^	0.001

## Data Availability

The original contributions presented in this study are included in the article/Supplementary Material. Further inquiries can be directed to the corresponding authors.

## Supplementary Materials

The following supporting information can be downloaded at: https://www.mdpi.com/article/10.3390/foods15050831/s1, Table S1. Nutritional composition of the bread food labels.

## Abbreviations

The following abbreviations are used in this manuscript:

CATA	Check-All-That-Apply
FAA	Free Amino Acids
TPC	Total Phenolic Content
PCA	Principal Component Analysis
HCA	Hierarchical Cluster Analysis
