# Supplementary material for: From Composition to Acceptance: Linking Nutritional, Structural and Sensory Attributes in Clean-Label Breads
Source: Foods. 2026 Mar 2;15(5):831. doi: 10.3390/foods15050831 (PMC12984663; doi:10.3390/foods15050831)
Supplement: Supplementary file 1 [file foods-15-00831-s001.zip › foods-4133178-supplementary.pdf]

Table S1. Nutritional composition of the bread food labels

Bread sample	Energy kcal/100 g	Fats		Carbohydrates		Dietary Fiber g/100	Protein g/100 g	Salt g/100 g	Vit E mg
		Total g/100 g	Saturates g/100 g	Total g/100 g	Sugars g/100 g				
**GB-A**	270	5.0	0.7	45.7	5.0	4.0	8.5	1.2	
**GB-B**	280	8.0	0.9	39	7.5	7.3	9.5	1.0	
**GB-C**	297	10	1.2	38	6.0	5.3	11	1.3	
**GB-D**	282	10	1.0	32.1	6.4	8.4	11.7	1.0	
**PB-E**	272	3.9	0.6	48.4	7.3	4.5	8.5	1.25	
**PB-F**	275	6.8	0.6	38	7.0	8.2	11.0	1.20	
**PB-G**	278	5.9	0.7	42	7.0	6.0	11.0	1.30	
**PB-H**	258	3.9	0.6	44.6	8.4	5.0	8.7	1.25	
**PB-I**	287	8.0	0.9	38	7.5	7.5	12	0.8	15
